# Postoperative opioid prescribing patients with diabetes: Opportunities for personalized pain management

**DOI:** 10.1371/journal.pone.0287697

**Published:** 2023-08-24

**Authors:** Alban Zammit, Jean Coquet, Jennifer Hah, Oualid el Hajouji, Steven M. Asch, Ian Carroll, Catherine M. Curtin, Tina Hernandez-Boussard

**Affiliations:** 1 Department of Medicine, Stanford University School of Medicine, Stanford, California, United States of America; 2 Institute for Computational & Mathematical Engineering, Stanford University, Stanford, California, United States of America; 3 Department of Anesthesiology, Perioperative, and Pain Medicine, Stanford University School of Medicine, Stanford, California, United States of America; 4 US Department of Veterans Affairs, Palo Alto Healthcare System, Palo Alto, California, United States of America; 5 Department of Surgery, VA Palo Alto Health Care System, Menlo Park, California, United States of America; 6 Department of Surgery, Stanford University School of Medicine, Stanford, California, United States of America; 7 Department of Biomedical Data Science, Stanford University, Stanford, California, United States of America; University of South Australia, AUSTRALIA

## Abstract

**Background:**

Opioids are commonly prescribed for postoperative pain, but may lead to prolonged use and addiction. Diabetes impairs nerve function, complicates pain management, and makes opioid prescribing particularly challenging.

**Methods:**

This retrospective observational study included a cohort of postoperative patients from a multisite academic health system to assess the relationship between diabetes, pain, and prolonged opioid use (POU), 2008–2019. POU was defined as a new opioid prescription 3–6 months after discharge. The odds that a patient had POU was assessed using multivariate logistic regression controlling for patient factors (e.g., demographic and clinical factors, as well as prior pain and opiate use).

**Findings:**

A total of 43,654 patients were included, 12.4% with diabetes. Patients with diabetes had higher preoperative pain scores (2.1 vs 1.9, p<0.001) and lower opioid naïve rates (58.7% vs 68.6%, p<0.001). Following surgery, patients with diabetes had higher rates of POU (17.7% vs 12.7%, p<0.001) despite receiving similar opioid prescriptions at discharge. Patients with Type I diabetes were more likely to have POU compared to other patients (Odds Ratio [OR]: 2.22; 95% Confidence Interval [CI]:1.69–2.90 and OR:1.44, CI: 1.33–1.56, respectively).

**Interpretation:**

In conclusion, surgical patients with diabetes are at increased risk for POU even after controlling for likely covariates, yet they receive similar postoperative opiate therapy. The results suggest a more tailored approach to diabetic postoperative pain management is warranted.

## Introduction

In the last decade, precision medicine has promised to revolutionize healthcare by allowing medical therapies to be tailored to the unique needs of each individual patient [1]. Precision medicine identifies patient factors such as genetic variants, comorbidities and social determinants of health that influence a treatment response [2]. Patient factors are particularly important for response to pain management, for example in oncology [3], with the elderly [4], or in postoperative settings [5]. The principle underlying precision medicine is that the same treatment may not be effective or optimal for all patients. For pain management, this means that prescribing the same pain medications at the same doses may not be appropriate for all patients. For example, previous work suggests that patients taking selective serotonin reuptake inhibitors (SSRIs) [6], the most prescribed class of antidepressants, achieve faster postoperative pain resolution when prescribed an active opioid versus an opioid requiring metabolic activation. Precision perioperative pain management considers the surgical event, clinical characteristics, demographics and psychosocial factors. Understanding different subpopulations’ pain management needs and associated pain outcomes is essential to advance precision prescribing.

Patients with diabetes are a unique population for which there is opportunity for precision prescribing. Diabetes changes nociceptive physiology resulting in increased hypersensitivity to pain and a weaker response to morphine, [7] particularly for neuropathic pain [8]. They report higher pain scores both preoperatively and postoperatively as compared to non-diabetic patients [9]. In a retrospective cohort of 583 patients undergoing elective cervical or lumbar spine surgery, diabetes was a significant preoperative risk factor for prolonged opioid use (POU) [10]. However, the analgesic effects of morphine for postoperative pain among patients with diabetes remains unclear [11–14]. Effective postoperative opioid prescribing for patients with diabetes is understudied at the population level leaving a gap in evidence and clinical guidelines.

In this study, we sought to quantify postoperative opioid prescribing and prolonged opioid prescriptions among diabetic patients as compared to other unique patient populations from a diverse healthcare center. The hypothesis was that prescription opioid strength, as measured by daily morphine milligram equivalents (MME), would not differ for patients with diabetes compared to non-diabetics. This work provides population-level evidence on current opioid prescribing practices at an academic institute and associated patient outcomes that can be used to guide new policies and precision medicine initiatives.

## Methods

### Study design

In this retrospective, observational study, Electronic Health Records (EHR) were used to identify adult surgical patients at a diverse health care center, which included an academic medical center, a community hospital, and primary and specialty care alliance, [15] between 2008 and 2019. The study followed the STROBE guidelines [16]. We received the HIPAA patient waiver allowing us to access and use the patient’s medical records for research purposes. This study received the approval from the Stanford Institutional Review Board (IRB).

### Data source and study participants

Participants included adults aged between 18 and 89 who underwent surgery at one of the healthcare facilities and were prescribed an opioid for postoperative pain management. The index date chosen for patients with records of multiple surgeries was the date of their first ever surgery. The list of opioids considered is given in S1 Table. *Current Procedural Terminology* (CPT) codes, *International Classification of Diseases*, *9th* and *10th Revision*, *Clinical Modification* (hereafter, ICD) codes, and *Procedures Coding Systems* (ICD10PCS) codes were used to catch patients who underwent a surgery. The *Clinical Classifications Software for Services and Procedures* (CCS-SP) was then used to categorize surgeries into 19 classes, which can be seen in S2 Table. The rates of procedures were comparable in patients with and without diabetes. This study used routinely captured medical data from electronic health records.

**Fig 1:**
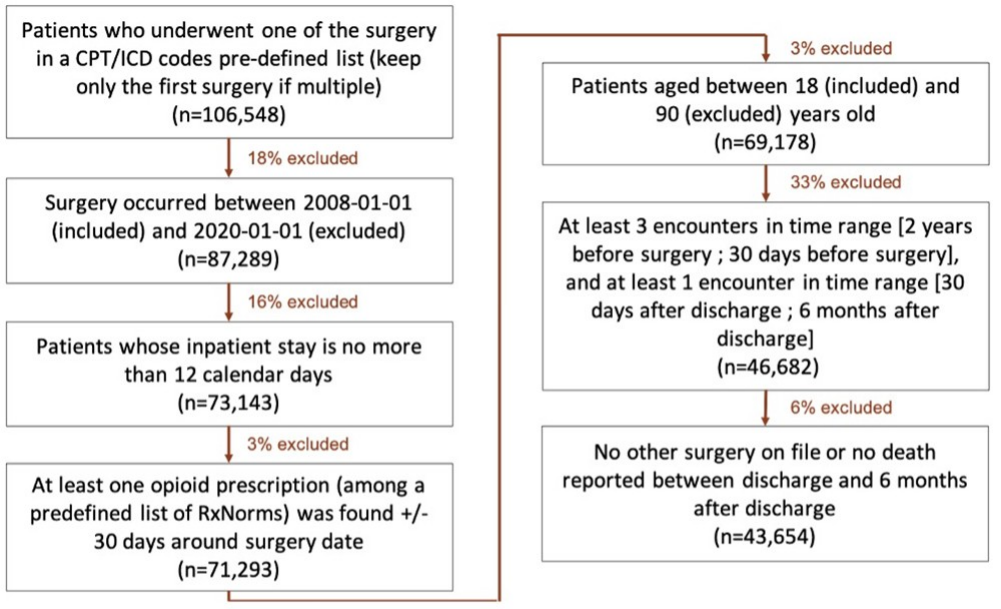
Flowchart of study cohort, 2008–2020.

Patients were excluded if their inpatient stay was greater than 13 days. This decreased inclusion of patients who had complications following surgery reducing heterogeneity of the patients’ postoperative course. Similarly, to properly study patient relationship to opioids after surgery, we excluded all patients who faced an external event or competing risk which might have forced them to take opioids independently of their postoperative pain management. Namely, we excluded patients who died or had another surgery less than 6 months after discharge of their first surgery. In addition, patient were excluded if they did not receive at least one opioid prescription following surgery (<3%). Finally, some patients only are seen once for a one-off surgery making studying their postoperative pain course impossible. Thus, we excluded patient with minimal follow up. The minimum numbers of encounters required on the different time windows before and after surgery is given in the flow chart (Fig 1) and was decided based upon the calibration curves available in S1 Fig.

### Study variables

Patient variables included Gender, Race/ethnicity (Non-Hispanic White, Hispanic, Non-Hispanic Asian, Non-Hispanic Black & Other), age at surgery, insurance type (Private, Medicaid, Medicare and Other), Charlson Comorbidity Index, and body mass index (BMI). We additionally included the number of tobacco packs smoked per day and volume of alcohol consumed per week as declared by the patient at the preoperative visit, and the preoperative pain score. Comorbidities were identified using ICD codes based on Clinical Classification Software [17]. Obesity was also identified using body mass index (BMI) from the EHR; patients with a BMI greater than 30 were categorized as obese.

Patient were stratified by documentation of diabetes any time before surgery using ICD-9 or ICD-10 codes. Patients were further stratified by Type I Diabetes (DM1) and Type II Diabetes (DM2). Clinical variables included the surgery category, as given by the CCS-SP codes, [18] anesthesia type (i.e., General, Monitored Anesthesia Care, Peripheral Nerve Block, Regional, Unknown/Other), patient’s *American Society of Anesthesiologists* (ASA) physical status at the preoperative visit and the length of stay [19].

We considered drug prescriptions on hand for each patient up to 6 months before surgery date. Opioid naivety before the surgery was defined as the absence of an opioid prescription up to 6 months before surgery. Our EHR system reconciles medications prescribed outside of our health system and medications that are self-reported by patients. Prescriptions were identified using RxNorm identifiers from the *Unified Medical Language* system (opioid prescriptions are listed in S1 Table). Opioid prescriptions were also converted to Morphine Milligram Equivalent (MME) associated following CDC recommendations [20].

For all variables included, missingness is reported in S3 Table. For missing data, imputations were needed for the adjusted odd ratio computations and consisted of the median for continuous variables and the most represented class for categorical variables.

### Outcome measures

The primary outcome of interest was prolonged opioid prescription. Many definitions for prolonged opioid use (POU) are used in the literature [21]. Based on discussion with clinicians, we define POU as a new opioid prescription starting 3 to 6 months after surgical discharge. A manual chart review on a random sample of 100 patients assessed this definition against a gold standard assigned by 5 physicians (S4 Table).

### Statistical analysis

Bivariate associations were measured using a Fisher’s exact test for categorical covariates, and using a one-way ANOVA for continuous covariates. Unadjusted odd ratio entailed by each variable against the outcome were computed with Fischer’s exact statistic, and a logistic regression was fitted with all study variables against the outcome to get adjusted odd-ratios that eliminate the confounding effects between covariates. All statistical tests were two-sided, and the null hypothesis was rejected if computed p-value was under the threshold of 0.05.

### Sensitivity analyses

Many different definitions for POU were compared, based on available definitions in a recent systematic review [21]. Based on these definitions, a manual chart review for 100 patients in the cohort was performed by five clinicians. These gold standards were compared with the different labels given by candidate definitions, and the definition used in this manuscript had the highest F1 score as explained in the methods section. Other metrics for our POU definition are given in S4 Table.

## Results

In the cohort of 43,654 surgical patients, a total of 5,417 (12,4%) patients with diabetes were identified (Table 1). Diabetic surgical patients were older (64.8 vs 56.8; p<0.001) and had lower proportions of non-Hispanics Whites (48.4% vs 63.5%; p<0.001) and private insurance (19.9% vs 38.0%; p<0.001) compared to those without diabetes. Diabetic patients also had higher preoperative pain scores, although not clinically significant, (2.1 vs 1.9; p<0.001) and a higher proportion of obese (28.3% vs 8.9%; p<0.001), depressed (18.1% vs 10.8%; p<0.001), and lower rates of opioid naïve (58.7% vs 68.6%; p<0.001) compared to those without diabetes. Regarding clinical characteristics, upon discharge a higher proportion of diabetic patients received Tramadol compared to non-diabetics (5.4% vs 4.8%; p<0.001), although there was no difference in average daily MMEs between patients with diabetes and non-diabetics (35.7 MME vs 35.8 MME; p = 0.924). Diabetic patients had higher rates of POU following surgery compared to non-diabetic patients, an asolite increase of 5% but a relative increase of almost 50% (17.7% vs 12.7%; p<0.001).

**Table 1:** Study patient characteristics stratified by diabetes diagnosis.

Variable		Total	No Diabetes Diagnosis	Diabetes Diagnosis	p-val
Total, No. (%)		43654	38237 (87.6%)	5417 (12.4%)	
Gender, No. (%)	Female	24230 (55.5)	21566 (56.4)	2664 (49.2)	<0.001
	Male	19424 (44.5)	16671 (43.6)	2753 (50.8)	
Age at Surgery (years), Mean (SD)		57.8 (16.0)	56.8 (16.3)	64.8 (12.0)	<0.001
Race/Ethnicity, No. (%)	Hispanic	5776 (13.2)	4752 (12.4)	1024 (18.9)	<0.001
	Non-Hispanic Asian	5468 (12.5)	4571 (12.0)	897 (16.6)	
	Non-Hispanic Black	1421 (3.3)	1139 (3.0)	282 (5.2)	
	Non-Hispanic White	26894 (61.6)	24274 (63.5)	2620 (48.4)	
	Other/Unknown	4095 (9.4)	3501 (9.2)	594 (11.0)	
Insurance Type, No. (%)	Medicaid	3108 (7.1)	2701 (7.1)	407 (7.5)	<0.001
	Medicare	16707 (38.3)	13765 (36.0)	2942 (54.3)	
	Other/Unknown	8230 (18.9)	7240 (18.9)	990 (18.3)	
	Private	15609 (35.8)	14531 (38.0)	1078 (19.9)	
Opioid Naive, No. (%)		29427 (67.4)	26246 (68.6)	3181 (58.7)	<0.001
2-year Charlson Score, Mean (SD)		0.4 (1.1)	0.4 (1.0)	0.8 (1.4)	<0.001
Body Mass Index (BMI), Mean (SD)		27.9 (6.4)	27.5 (6.1)	30.8 (7.3)	<0.001
Obesity Diagnosis, No. (%)		4923 (11.3)	3388 (8.9)	1535 (28.3)	<0.001
Depression Diagnosis, No. (%)		5120 (11.7)	4137 (10.8)	983 (18.1)	<0.001
HbA1c Percentage, Mean (SD)		6.1 (1.2)	5.6 (0.8)	6.8 (1.3)	<0.001
ASA^1^ Status, Mean (SD)		2.3 (0.7)	2.2 (0.7)	2.7 (0.6)	<0.001
Preoperative Pain Score, Mean (SD)		2.0 (2.5)	1.9 (2.5)	2.1 (2.6)	<0.001
Alcohol Volume per Week (Oz), Mean (SD)		0.9 (3.2)	1.0 (3.3)	0.5 (2.0)	<0.001
Nb. Tobacco Packs per Day, Mean (SD)		0.2 (0.6)	0.2 (0.6)	0.3 (0.6)	<0.001
**Clinical Characteristics**					
Length of Stay (days), Mean (SD)		2.8 (2.8)	2.7 (2.7)	3.5 (3.0)	<0.001
Anesthesia Type, No. (%)	Monitored Anesthesia Care	1787 (4.1)	1597 (4.2)	190 (3.5)	0.011
	Other/Unknown	6018 (13.8)	5274 (13.8)	744 (13.7)	
	General	34045 (78.0)	29820 (78.0)	4225 (78.0)	
	Regional	1804 (4.1)	1546 (4.0)	258 (4.8)	
Discharge Daily MME (mg), Mean (SD)		35.8 (111.3)	35.8 (118.3)	35.7 (33.7)	0.924
Outpatient Opioid Type, No. (%)	Codeine	893 (2.0)	782 (2.0)	111 (2.0)	0.023
	Hydrocodone	15144 (34.7)	13273 (34.7)	1871 (34.5)	
	Hydromorphone	589 (1.3)	510 (1.3)	79 (1.5)	
	Morphine	427 (1.0)	368 (1.0)	59 (1.1)	
	Other/Unknown	5495 (12.6)	4888 (12.8)	607 (11.2)	
	Oxycodone	18973 (43.5)	16574 (43.3)	2399 (44.3)	
	Tramadol	2133 (4.9)	1842 (4.8)	291 (5.4)	
Prolonged Opioid User, No. (%)		5819 (13.3)	4859 (12.7)	960 (17.7)	<0.001

^1^American Society of Anesthesiologists

Patients were further stratified by Type I Diabetes (DM1) and Type II Diabetes (DM2) (Table 2). DM1 patients had lower preoperative pain scores compared to DM2 patients (1.7 vs 2.1; p = 0.003) and had similar discharge daily MMEs prescribed (32.9 MME vs 35.9 MME; p = 0.105). However, 24.4% of DM1 patients requested additional opioids 3–6 months following surgery (i.e., POU) compared to only 17.3% of DM2 patients (p = 0.003).

**Table 2:** Patient and clinical characteristics, stratified by Diabetes Type I (DM Type I) and Type 2 (DM Type II).

Variable		Total	DM Type I	DM Type II	p-val
Total, No. (%)		5417	291 (5.4%)	5126 (94.6%)	
Gender, No. (%)	Female	2664 (49.2)	147 (50.5)	2517 (49.1)	0.683
	Male	2753 (50.8)	144 (49.5)	2609 (50.9)	
Age at Surgery (years), Mean (SD)		64.8 (12.0)	57.4 (15.5)	65.2 (11.7)	<0.001
Race/Ethnicity, No. (%)	Hispanic	1024 (18.9)	47 (16.2)	977 (19.1)	0.001
	Non-Hispanic Asian	897 (16.6)	28 (9.6)	869 (17.0)	
	Non-Hispanic Black	282 (5.2)	12 (4.1)	270 (5.3)	
	Non-Hispanic White	2620 (48.4)	173 (59.5)	2447 (47.7)	
	Other/Unknown	594 (11.0)	31 (10.7)	563 (11.0)	
Insurance Type, No. (%)	Medicaid	407 (7.5)	21 (7.2)	386 (7.5)	<0.001
	Medicare	2942 (54.3)	129 (44.3)	2813 (54.9)	
	Other/Unknown	990 (18.3)	55 (18.9)	935 (18.2)	
	Private	1078 (19.9)	86 (29.6)	992 (19.4)	
Opioid Naive, No. (%)		3181 (58.7)	165 (56.7)	3016 (58.8)	0.510
2-year Charlson Score, Mean (SD)		0.8 (1.4)	1.0 (1.4)	0.8 (1.4)	0.014
Body Mass Index (BMI), Mean (SD)		30.8 (7.3)	28.4 (6.3)	31.0 (7.3)	<0.001
Obesity Diagnosis, No. (%)		1535 (28.3)	63 (21.6)	1472 (28.7)	0.011
Depression Diagnosis, No. (%)		983 (18.1)	65 (22.3)	918 (17.9)	0.067
HbA1c Percentage, Mean (SD)		6.8 (1.3)	7.2 (1.5)	6.8 (1.3)	<0.001
ASA Status, Mean (SD)		2.7 (0.6)	2.7 (0.6)	2.7 (0.6)	0.253
Preoperative Pain Score, Mean (SD)		2.1 (2.6)	1.7 (2.2)	2.1 (2.7)	0.003
Alcohol Volume per Week (Oz), Mean (SD)		0.5 (2.0)	0.7 (2.0)	0.5 (2.0)	0.028
Nb. Tobacco Packs per Day, Mean (SD)		0.3 (0.6)	0.2 (0.5)	0.3 (0.6)	0.036
**Clinical Characteristics**					
Length of Stay (days), Mean (SD)		3.5 (3.0)	3.4 (3.3)	3.6 (2.9)	0.475
Anesthesia Type, No. (%)	Monitored Anesthesia Care	190 (3.5)	18 (6.2)	172 (3.4)	0.063
	Other/Unknown	744 (13.7)	43 (14.8)	701 (13.7)	
	General	4225 (78.0)	215 (73.9)	4010 (78.2)	
	Regional	258 (4.8)	15 (5.2)	243 (4.7)	
Discharge Daily MME (mg), Mean (SD)		35.7 (33.7)	32.9 (30.6)	35.9 (33.9)	0.105
Outpatient Opioid Type, No. (%)	Codeine OR Hydromorphone OR Morphine^1^	249 (4.6)	14 (4.8)	235 (4.6)	0.011
	Hydrocodone	1871 (34.5)	98 (33.7)	1773 (34.6)	
	Other/Unknown	607 (11.2)	51 (17.5)	556 (10.8)	
	Oxycodone	2399 (44.3)	113 (38.8)	2286 (44.6)	
	Tramadol	291 (5.4)	15 (5.2)	276 (5.4)	
Prolonged Opioid User, No. (%)		960 (17.7)	71 (24.4)	889 (17.3)	0.003

^1^Outpatient Opioid Types Codeine, Hydromorphone and Morphine were collapsed into a single category to comply with privacy regulations that prevent the reporting of groups with less than 10 participants

**Fig 2:**
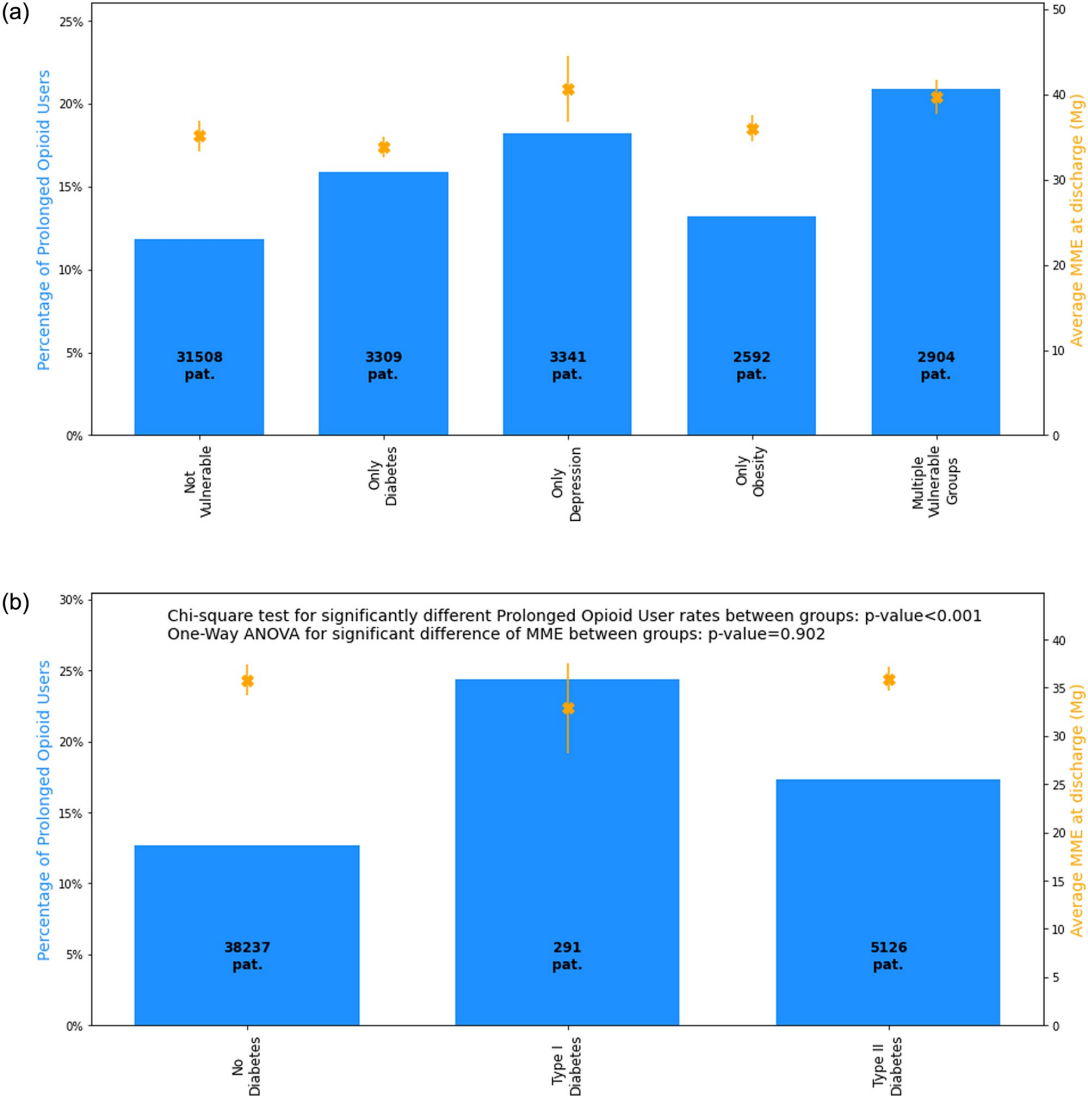
Percent of prolonged opioid users and average Daily MME at Discharge, Stratified by Vulnerable Patients (A) and by Diabetes Type (B).

Fig 2(A) shows no difference in MMEs across different patient characteristics, diabetic, obese, or depressed (p>0.05), however these populations had higher proportions of patients with POU compared to other patients (p<0.001). Similarly, Fig 2(B) shows that DM1 and DM2 patients received similar MMEs prescriptions at discharge (p<0.902) yet were more likely to be POU (p<0.001).

Predictors of POU are presented in Table 3. DM1 patients have a higher odds of becoming POU (OR: 2.633; CI: 1.970–3.519) followed by DM2 patients (OR: 1.56; CI: 1.430–1.707). S5 Table presents all variables positively predictive of POU.

**Table 3:** Predictors of prolonged opioid use after surgery (vs. no prolonged opioid use).

Variables		Unadjusted		Adjusted	
		Odd Ratio (CI)	p-val	Odd Ratio (CI)	p-val
**Age Group (ref: 40–75—N = 31463)**	19–39 (N = 6562)	0.919 (0.849–0.995)	0.037	0.955 (0.874–1.043)	0.307
	>75 (N = 5629)	0.859 (0.787–0.936)	<0.001	0.842 (0.765–0.928)	<0.001
**Gender (ref: Female—N = 24230)**	Male (N = 19424)	0.832 (0.786–0.880)	<0.001	0.967 (0.906–1.032)	0.311
**Race/Ethnicity (ref: Non-Hispanic White—N = 26894)**	Hispanic (N = 5776)	1.197 (1.105–1.297)	<0.001	1.081 (0.991–1.180)	0.08
	Non-Hispanic Asian (N = 5468)	0.964 (0.883–1.052)	0.415	0.847 (0.771–0.930)	<0.001
	Other/Unknown (N = 4095)	0.801 (0.721–0.889)	<0.001	0.806 (0.723–0.900)	<0.001
	Non-Hispanic Black (N = 1421)	1.713 (1.499–1.958)	<0.001	1.499 (1.301–1.728)	<0.001
**Diabetes (ref: No Diabetes Diagnosis—N = 38237)**	Type II (N = 5126)	1.441 (1.333–1.559)	<0.001	1.562 (1.430–1.707)	<0.001
	Type I (N = 291)	2.217 (1.694–2.902)	<0.001	2.633 (1.970–3.519)	<0.001
**Depression (ref: No Depression Diagnosis—N = 38534)**	Depression Diagnosis (N = 5120)	1.780 (1.652–1.918)	<0.001	1.429 (1.318–1.549)	<0.001
**Obesity (ref: No Obesity Diagnosis—N = 38731)**	Obesity Diagnosis (N = 4923)	1.308 (1.206–1.418)	<0.001	1.294 (1.171–1.429)	<0.001
**Not Opioid Naive (ref: Opioid Naive—N = 29427)**	Not Opioid Naive (N = 14227)	2.439 (2.307–2.580)	<0.001	1.899 (1.789–2.016)	<0.001
**Insurance Type (ref: Medicare—N = 16707)**	Private (N = 15609)	0.907 (0.849–0.969)	0.004	0.944 (0.875–1.018)	0.135
	Other/Unknown (N = 8230)	1.261 (1.171–1.359)	<0.001	1.211 (1.116–1.314)	<0.001
	Medicaid (N = 3108)	1.236 (1.110–1.375)	<0.001	1.052 (0.935–1.184)	0.401

## Discussion

Optimal postoperative pain management for patients with diabetes remains unclear. In this study of real-world care, we found uniform postoperative opioid prescribing regardless of diabetes diagnosis. Specifically, in this large multiethnic population there was no difference in discharge opioid MMEs between patients with and without diabetes even though diabetic patients presented with known pain risk factors, including higher rates of previous opioid use, depression, and preoperative pain scores. Following surgery patients with diabetes had higher rates of POU compared to non-diabetic patients. Diabetic patients have unique physiology and heightened pain, which argues the need for more tailored postoperative pain management. This study provides considerations for postoperative opioid prescribing to patients with diabetes using real world data.

People with diabetes have several physiologic changes that make pain management more complex. Multimodal analgesia combines analgesic medications and techniques (e.g., epidural analgesia) targeting different pain mechanisms with the goal of synergistic pain relief. With concurrent use of non-opioid pain medications such as acetaminophen, [22] non-steroidal anti-inflammatory drugs (NSAIDs), [23] and gabapentinoids; [24] reductions in postoperative opioid consumption have often been reported in the first 24 to 48 hours after surgery [22, 25, 26]. However, there is limited data regarding the long-term opioid-sparing outcomes of these medications after hospital discharge with prolonged treatment in the weeks to months after surgery spanning the subacute to chronic postoperative phases. Further, these commonly prescribed non-opioid postoperative pain medications may be particularly hazardous for patients with diabetes. For example, NSAIDS use among patients with diabetes may compound risk of acute kidney injury, progression of chronic kidney disease, hyperkalemia, and papillary necrosis [27]. For patients with diabetes with chronic kidney disease, gabapentin requires dose adjustments for reduced kidney function. Given the increased risk of opioid overdose and respiratory depression among patients with medical comorbidities, care should be taken when adding gabapentin or pregabalin to the postoperative pain management regimen for patients with diabetes [28, 29]. Future research to determine the postoperative opioid-sparing effects of different analgesics among patients with diabetes may improve postoperative pain care for these patients.

Preoperative opioid use is one of the most significant determinants of POU. In this study, patients with diabetes had a higher incidence of preoperative opioid use. However, our study adds that even after accounting for preoperative opioid use, DMI or DMII is an independent risk factor for POU. These findings, if confirmed in other data, provide several next steps. For example, we found providers generally are not accounting for individual risk factors with postoperative opioid prescribing; with every patient prescribed uniform doses. Furthermore, within this academic setting, opioid rates have remained fairly constant over this time period, as we have previously shown [30]. Given the increased risk of POU in patient with diabetes, education and clinical support tools exist and could be further refined to alert providers on a more tailored plan for patients at risk, including diabetic patients [31].

There were also important differences in opioid-related outcomes between DMI and DMII surgical patients. While in general diabetic patients had higher rates of POU, DMI patients had significantly higher POU rates compared to DMII patients, yet again there was no difference in the quantity of MMEs received at discharge. Specifically, we found DMII patients had a 2-fold increased risk for obtaining a new opioid prescription 3 to 6 months after surgery. This was a surprising finding and highlights the need for closer monitoring of DMII patients following surgery. The literature is clear that pain is a common adverse outcome of diabetes. But most of this evidence has not compared the pain experience between DMI and DMII. However, there is some animal evidence that the nociceptive pathways in DMI and DMII differ [32]. This finding highlights the need for population studies to identify important patient factors within patients with diabetes that impact pain outcomes.

Both DM1 and DMII patients are characterized by chronic elevations of blood glucose, and the microvascular sequelae associated with this including impaired wound healing, neuropathy retinopathy, and nephropathy. However, DMI is typically acquired early in life while DMII is a disease of the aging adult. We hypothesize that the increased rate of POU among those with DMI may reflect the more prolonged exposure to the metabolic disturbance in these patients, analogous to renal and ocular damage that is more common in DMI compared to DM2 after controlling for age [33, 34]. Future studies should evaluate if years of exposure (i.e., time from diagnosis) better predicts the increased odds of POU than specific diagnosis (DMI vs DMII). Similarly, the degree of hyperglycemia over time may partially mediate this effect, and the interaction of degree of elevation in hemoglobin A1C and time over which the patients has been exposed to that hyperglycemia (time since diagnosis) may ultimately prove better at predicting individual risk of POU than the specific diagnosis [35]. These results suggest that in the same way we appreciate diabetes as a systemic condition impairing the vascular system, the peripheral nervous system, the kidneys and retinas, we need to appreciate an effect on pro-nociceptive components of the sensory nervous system.

Prior research reported diabetes as a risk factor for POU [10, 36–40]. However, most studies did not differentiate outcomes between DMI and DMII patients, and our findings further highlight disparities among diabetic subgroups warranting further investigation. Future granular research detailing disparities in surgical management among patients with diabetes and non-diabetics is warranted to understand the increased risk of POU among patients with diabetes. Future research to define the mediating effects of glycosylated hemoglobin in the acute, subacute and remote postoperative phases are needed to characterize the mechanisms underlying increased postoperative opioid needs among patients with diabetes. We extend these findings by characterizing POU 3–6 months after a diverse array of operations to characterize the unique patient-specific risk of preoperative DMI and DMII in the development of POU.

This study has limitations. The Northern California-based cohort was multisite and diverse but may not be generalizable across the United States diabetic population. However, compared to the US statistics, [41] the racial/ethnic distribution in the cohort is similar to the NHANES with the except of Black patients. Care fragmentation may result in missing outside opioid data, but the EHR system reconciles medications prescribed outside of the health system and medications that are self-reported by patients, thus mitigating this limitation. This was a retrospective, observational study and there are other potential confounding factors that cannot be measured. However, substantial efforts were made to capture many confounding variables, such as factors prior surgery, length of hospital admission, and psychosocial factors. Finally, patients with diabetes were identified using only diagnostic codes. Patients coming to the center only for surgical treatment may lack exhaustive comorbidity documentation, including diabetes codes. However, diabetes rates in the population are aligned with rates reported in the NHANES cohort and as DM is an important surgical risk factor, it is well documented for procedures.

In conclusion, in a multiethnic cohort of diabetic surgical patients, nearly 20% requested an additional opioid prescription three months after surgery. After controlling for important covariates, patients with DMI had over a two-fold risk of POU. In addition, our findings highlight a lack of tailored opioid prescribing for this vulnerable population. These finding present evidence that may guide opioid prescribing among diabetic surgical patients to improve patient outcomes in vulnerable populations.

## Supporting information

S1 Table List of RxNorms considered as opioid medications. (DOCX)

S2 Table Surgery classes of study patients, stratified by diabetes diagnosis. (DOCX)

S3 Table Report of missing data for each variable. (DOCX)

S4 Table Manual assessment of the chosen definition for prolonged opioid use. (DOCX)

S5 Table All variables with significantly predictive odd ratio of prolonged opioid use. (DOCX)

S1 Fig Calibration curves to choose the minimum numbers of encounters required before and after surgery for patients to be included in the cohort. (PDF)

S1 Checklist STROBE statement—Checklist of items that should be included in reports of observational studies. (DOCX)
