# Supplementary material for: Postoperative opioid prescribing patients with diabetes: Opportunities for personalized pain management
Source: PLoS One. 2023 Aug 24;18(8):e0287697. doi: 10.1371/journal.pone.0287697 (PMC10449216; doi:10.1371/journal.pone.0287697)
Supplement: S1 Table — (DOCX) [file pone.0287697.s001.docx]

**SUPPLEMENTARY MATERIAL**

**eTable 1. List of RxNorms considered as opioid medications**

| **Opioid molecule** | **RxNorm** |
| --- | --- |
| buprenorphine | 1819 |
| butorphanol | 1841 |
| codeine | 2670 |
| dextromoramide | 3290 |
| dezocine | 22713 |
| dihydrocodeine | 23088 |
| fentanyl | 4337 |
| hydrocodone | 5489 |
| hydromorphone | 3423 |
| methadone | 6813 |
| meperidine | 6754 |
| meptazinol | 6761 |
| morphine | 7052 |
| nalbuphine | 7238 |
| opium | 7676 |
| oxycodone | 7804 |
| oxymorphone | 7814 |
| papaveretum | 7894 |
| pentazocine | 8001 |
| phenazocine | 8119 |
| pirinitramide | 8354 |
| propoxyphene | 8785 |
| tapentadol | 787390 |
| tilidine | 10597 |
| tramadol | 10689 |
