# Supplementary material for: Postoperative opioid prescribing patients with diabetes: Opportunities for personalized pain management
Source: PLoS One. 2023 Aug 24;18(8):e0287697. doi: 10.1371/journal.pone.0287697 (PMC10449216; doi:10.1371/journal.pone.0287697)
Supplement: S2 Table — (DOCX) [file pone.0287697.s002.docx]

**eTable 2. Surgery Classes of Study Patients, stratified by Diabetes Diagnosis**

| **Variable** | | **Total** | **No Diabetes Diagnosis** | **Diabetes** | **p-val** |
| --- | --- | --- | --- | --- | --- |
| Total, No. (%) | | 43654 | 38237 (87.6%) | 5417 (12.4%) |  |
| Surgery Category, No. (%) | Appendectomy | 831 (1.9) | 772 (2.0) | 59 (1.1) | <0.001 |
|  | CABG | 807 (1.8) | 591 (1.5) | 216 (4.0) |  |
|  | Colorectal Resection | 2365 (5.4) | 2088 (5.5) | 277 (5.1) |  |
|  | Distal Radius Fracture | 664 (1.5) | 603 (1.6) | 61 (1.1) |  |
|  | Excision/Lysis Peritubal Adhesions | 1268 (2.9) | 1023 (2.7) | 245 (4.5) |  |
|  | Hysterectomy (Vaginal/Abdominal) | 3427 (7.9) | 3103 (8.1) | 324 (6.0) |  |
|  | Inguinal Hernia Repair | 3024 (6.9) | 2618 (6.8) | 406 (7.5) |  |
|  | Knee Replacement | 4291 (9.8) | 3519 (9.2) | 772 (14.3) |  |
|  | Oophorectomy | 1243 (2.8) | 1162 (3.0) | 81 (1.5) |  |
|  | Other Hand | 469 (1.1) | 428 (1.1) | 41 (0.8) |  |
|  | Partial Excision of Bone | 4934 (11.3) | 4532 (11.9) | 402 (7.4) |  |
|  | Spinal Fusion | 4305 (9.9) | 3685 (9.6) | 620 (11.4) |  |
|  | Fracture Hip or Femur | 1303 (3.0) | 1128 (3.0) | 175 (3.2) |  |
|  | Frac Lower Extremity | 1655 (3.8) | 1483 (3.9) | 172 (3.2) |  |
|  | Colecystectomy | 2807 (6.4) | 2380 (6.2) | 427 (7.9) |  |
|  | Laminectomy | 3672 (8.4) | 3256 (8.5) | 416 (7.7) |  |
|  | Mastectomy | 2257 (5.2) | 2114 (5.5) | 143 (2.6) |  |
|  | Prostatectomy | 2323 (5.3) | 1988 (5.2) | 335 (6.2) |  |
|  | Thoracotomy | 2009 (4.6) | 1764 (4.6) | 245 (4.5) |  |
