# Supplementary material for: Postoperative opioid prescribing patients with diabetes: Opportunities for personalized pain management
Source: PLoS One. 2023 Aug 24;18(8):e0287697. doi: 10.1371/journal.pone.0287697 (PMC10449216; doi:10.1371/journal.pone.0287697)
Supplement: S3 Table — (DOCX) [file pone.0287697.s003.docx]

**eTable 3. Report of missing data for each variable**

| **Variable** | **Nunmber of missing values** | **Percentage of missing values** |
| --- | --- | --- |
| ASA Status | 5171 | 11.8 |
| HbA1c Percentage | 32649 | 74.8 |
| Age at Surgery | 0 | 0 |
| Alcohol Volume per Week | 125 | 0.3 |
| Anesthesia Type | 1233 | 2.8 |
| Body Mass Index | 420 | 1 |
| 2-year Charlson Score | 0 | 0 |
| Diabetes Diagnosis | 0 | 0 |
| Depression Diagnosis | 0 | 0 |
| Obesity Diagnosis | 0 | 0 |
| Gender | 0 | 0 |
| Length of Stay | 0 | 0 |
| Insurance Type | 0 | 0 |
| Opioid Naive | 0 | 0 |
| Discharge Daily MME | 7089 | 16.2 |
| Outpatient Opioid Type | 5050 | 11.6 |
| Preoperative Pain Score | 9409 | 21.6 |
| Prolonged Opioid User | 0 | 0 |
| Race/Ethnicity | 0 | 0 |
| Surgery Category | 0 | 0 |
| Nb. Tobacco Packs per Day | 125 | 0.3 |
