# Supplementary material for: Postoperative opioid prescribing patients with diabetes: Opportunities for personalized pain management
Source: PLoS One. 2023 Aug 24;18(8):e0287697. doi: 10.1371/journal.pone.0287697 (PMC10449216; doi:10.1371/journal.pone.0287697)
Supplement: S4 Table — (DOCX) [file pone.0287697.s004.docx]

**eTable 4. Manual Assessment of the Chosen Definition for Prolonged Opioid Use**

|  | Gold Standard: Prolonged Opioid User | Gold Standard: Not Prolonged Opioid User |
| --- | --- | --- |
| Label with Definition: Prolonged Opioid User | 14 | 3 |
| Label with Definition: Not Prolonged Opioid User | 2 | 81 |
| **Accuracy: 0.950, Precision: 0.824; Recall: 0.875; F1-score: 0.848** | | |
