# Supplementary material for: Postoperative opioid prescribing patients with diabetes: Opportunities for personalized pain management
Source: PLoS One. 2023 Aug 24;18(8):e0287697. doi: 10.1371/journal.pone.0287697 (PMC10449216; doi:10.1371/journal.pone.0287697)
Supplement: S5 Table — (DOCX) [file pone.0287697.s005.docx]

**eTable 5. All variables with significantly predictive odd ratio of prolonged opioid use**

| **Variable** | **Adjusted Odd Ratio** | **Confidence Interval** | **P-value** |
| --- | --- | --- | --- |
| Type I Diabetic | 2.633 | (1.970-3.519) | <0.001 |
| Surgery Category - Mastectomy | 2.625 | (2.302-2.993) | <0.001 |
| Not Opioid Naïve | 1.899 | (1.789-2.016) | <0.001 |
| Type II Diabetic | 1.562 | (1.430-1.707) | <0.001 |
| Race/Ethnicity - Non-Hispanic Black | 1.499 | (1.301-1.728) | <0.001 |
| Depression Diagnosis | 1.429 | (1.318-1.549) | <0.001 |
| Outpatient Opioid Type - Morphine | 1.386 | (1.096-1.754) | 0.007 |
| Obesity Diagnosis | 1.294 | (1.171-1.429) | <0.001 |
| Insurance Type - Other/Unknown | 1.211 | (1.116-1.314) | <0.001 |
| Anesthesia Type - Other/Unknown | 1.189 | (1.088-1.299) | <0.001 |
| ASA Status | 1.140 | (1.083-1.200) | <0.001 |
| 2-year Charlson Score | 1.116 | (1.091-1.142) | <0.001 |
| Nb. Tobacco Packs per Day | 1.086 | (1.038-1.136) | <0.001 |
| Length of Stay | 1.074 | (1.062-1.087) | <0.001 |
| Preoperative Pain Score | 1.062 | (1.048-1.075) | <0.001 |
