# Supplementary material for: Postoperative opioid prescribing patients with diabetes: Opportunities for personalized pain management
Source: PLoS One. 2023 Aug 24;18(8):e0287697. doi: 10.1371/journal.pone.0287697 (PMC10449216; doi:10.1371/journal.pone.0287697)

eFigure 1. Calibration curves to choose the minimum numbers of encounters required before and after surgery for patients to be included in the cohort

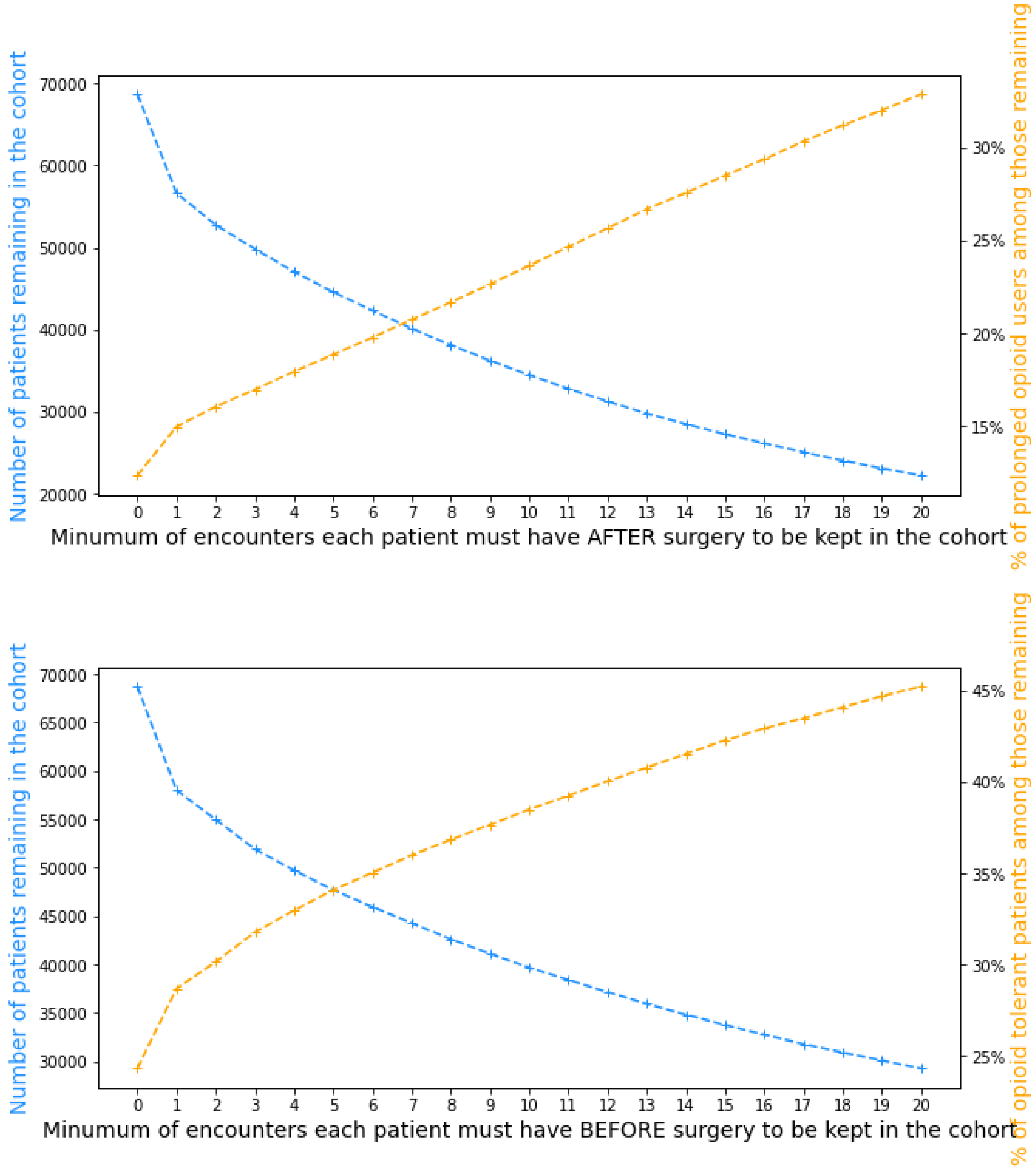

Supplement: S1 Fig — (PDF) [file pone.0287697.s006.pdf]
